# Relationship between Commuting Distance Using Public Transportation and the Risk of SARS-CoV-2 Infection in Healthcare Workers in Japan: A Cross-sectional Study

**DOI:** 10.24546/0100495532

**Published:** 2025-04-02

**Authors:** RYOHEI HASHIMOTO, KANAKO TERAMOTO, SOSHIRO OGATA, KOJI IIHARA, YOSHIHARU MIYATA, YOICHI KUREBAYASHI, KUNIHIRO NISHIMURA

**Affiliations:** 1 Department of Preventive Medicine and Epidemiology, National Cerebral and Cardiovascular Center, Osaka, Japan; 2 Department of Artificial Intelligence and Digital Health Science, Kobe University Graduate School of Medicine, Kobe, Japan; 3 Department of Biostatistics, National Cerebral and Cardiovascular Center, Osaka, Japan; 4 Department of Neurosurgery, National Cerebral and Cardiovascular Center, Osaka, Japan

**Keywords:** SARS-CoV-2, Seroprevalence, Healthcare Workers, Public Transportation, Commuting

## Abstract

This study aimed to explore the association between commuting distance using public transportation and the severe acute respiratory syndrome coronavirus 2 (SARS-CoV-2) antibody-positivity among medical staff at a cardiovascular medical institution in Japan. Information on the commuting distance using public transportation, grouped into none, short (<11.3 km; median), and long (≥11.3 km); demographics; and the the coronavirus disease 2019 (COVID-19) exposure were cross-sectionally collected from 956 employees in June 2022. SARS-CoV-2 antibody-positivity was defined based on serological tests for the nucleocapsid protein antigen. Among all participants (mean age 36 years; 68.6% female), 118 (12.3%) had SARS-CoV-2 antibody-positivity. Participants with long commuting distances were more likely to have ≥3 household members. Compared with non-use of public transportation, neither short nor long commuting distances by public transportation were associated with antibody-positivity (adjusted odds ratio 1.18 [95% confidence interval 0.70–1.98] and 1.62 [0.97–0.97–2.71], respectively). In participants with ≤2 household members (n = 706 [73.8%]; mean age 37 years; 72.4% female), a long commuting distance was associated with SARS-CoV-2 positivity compared with non-use of public transportation (1.98 [1.02–3.84]). In conclusion, commuting distance using public transportation was not associated with SARS-CoV-2 antibody-positivity in general; however, it may be relevant among healthcare workers with fewer household members.

## INTRODUCTION

The coronavirus disease 2019 (COVID-19) pandemic, caused by the severe acute respiratory syndrome coronavirus 2 (SARS-CoV-2), was a major global health problem that significantly impacted healthcare, economies, and everyday life (1). One of the features of this pandemic is the substantial prevalence of asymptomatic cases (2). A comprehensive systematic review indicates that nearly 40% of individuals infected with the virus exhibit no noticeable symptoms (3). This high proportion of asymptomatic cases complicates efforts to understand virus transmission, as these individuals can unknowingly spread the virus to others. Therefore, public transport is a key factor that is possibly related to the spread of SARS-CoV-2 (4–7). Several studies have highlighted the elevated risk of virus transmission among users of public transport systems, such as buses and trains (8–11). Although understanding that these dynamics is crucial, particularly in busy urban areas where public transportation is commonly used for daily commuting (12), much of the existing literature relies on self-reported, internet-based surveys, which may not be entirely accurate, especially for patients with asymptomatic cases.

In Japan, SARS-CoV-2 antibody testing has been conducted to understand the spread of the virus among different populations (13). Remarkably, antibody tests conducted among healthcare workers revealed that 42% of cases were overlooked, highlighting that traditional diagnostic methods, such as polymerase chain reaction tests and symptom-based screening, may not capture the true spread of the pandemic (14). In this study, we focused on antibody testing against the nucleocapsid (N) protein of the SARS-CoV-2 virus, which detects past infections irrespective of vaccination status (15). This method is particularly useful in identifying previously undetected cases in patients who are at significant risk owing to their job exposure or commuting patterns.

To gain new insights with the potential to inform public health guidelines and intervention strategies to tackle the spread of the virus, we performed a retrospective analysis, examining the association between commuting distance and SARS-CoV-2 infection in 2022 among hospital staff.

In this study, we also examined the association between household size and the relationship between commuting patterns and SARS-CoV-2 infection risk. Previous research has identified household size as a significant factor in virus transmission, with larger households associated with an increased risk of infection (16). However, there is little indication whether the same trend would be found in Japan as in abroad because of the declining birthrate and the shift to nuclear families. By conducting a subgroup analysis based on the number of household members, we aimed to account for the potential confounding effect of household transmission on the relationship between commuting distance and SARS-CoV-2 antibody-positivity. This approach allows us to better understand how commuting patterns may differently relate to individuals from different household sizes.

## MATERIALS AND METHODS

### Study design and participants

This research utilized a cross-sectional study design to investigate the relationship between commuting patterns and COVID-19 risk among healthcare workers in Osaka, Japan. The study was part of an observational study conducted in the National Cerebral and Cardiovascular Center (NCVC), a major urban medical and research institution, to monitor the presence of SARS-CoV-2 antibodies among employees during the COVID-19 pandemic.

The study involved 1,206 employees of the NCVC, including full-time and part-time medical professionals and administrative and support staff. Participants had to meet all three of the following inclusion criteria: 1) be an employee of the NCVC, 2) provide written informed consent for participation, and 3) be eligible for the employee medical examination of the first half of the 2022 annual medical checkup. Exclusion criteria for the study consisted of any of the following conditions: 1) individuals on long-term medical leave, parental leave, or extended vacation deemed unsuitable for the study by the principal investigator; 2) those eligible but not participating in the employee medical examination; and 3) those who had retired from the fiscal year 2021 onward and could not be reached for participation. A survey for this study was conducted through a web or written questionnaire.

This study was conducted following ethical guidelines and was approved by the Ethics Committee of the National Cerebral and Cardiovascular Center (R20102-5).

### SARS-CoV-2 antibody testing

Blood samples were collected from the participants during their medical examination for antibody testing. Antibodies were measured using the Elecsys Anti-SARS-CoV-2 RUO (Roche Diagnostics, Basel, Switzerland) (including immunoglobulin G). This test employs a modified double-antigen sandwich immunoassay that utilizes recombinant N protein to quantify antibodies. While antibodies against the spike protein indicate both infection and vaccination, antibodies against the N protein specifically indicate natural infection. The antibody tests were conducted following the manufacturer’s protocol. A positive result was defined as a cutoff index of ≥1.0. All tests were conducted at the testing department of the NCVC.

### Statistical analysis

Participants were categorized into three commuting distance groups: none (no use of public transportation), short (median 4.7, min–max [1.0–11.2] km), and long (median 21.3, min–max [11.3–97.9] km), based on the median value of the public transportation users. Baseline characteristics were presented across commuting distance groups, with medians (interquartile ranges, IQRs) for continuous variables and numbers (percentages, %) for categorical variables. Multivariate logistic regression models were used to calculate odds ratios (ORs) and 95% confidence intervals (CIs) of having positive antibody test results, with no use of public transportation as the reference. A linear trend test was used to determine the trend in OR across the commuting distance groups, with a p-value for trend reported.

Associations between commuting distance and antibody-positivity rate were adjusted for potential confounders. Confounders were selected based on their established or hypothesized associations with both commuting patterns and SARS-CoV-2 infection risk, as identified in the literature (16, 17). Specifically, we adjusted for age, sex, and body mass index (BMI) as basic demographic factors known to influence SARS-CoV-2 infection (17). We categorized BMI into three groups: underweight for BMI <18.5 kg/m^2^_,_ healthy weight for BMI ≥18.5 kg/m^2^ but <25 kg/m^2^, and overweight for BMI ≥25 kg/m^2^.

In this analysis, three models were employed: Model 1 was initially adjusted for potential confounding by basic participant background factors (age, sex, and BMI). To determine the role of commuting distance independently of other major viral transmission factors, Model 2 was adjusted for job type (categorized into three groups: high-risk exposure occupations include doctors and nurses; middle-risk exposure occupations include co-medical and medical affairs; and low-risk exposure occupations include administrative staff of the hospital, researchers, and service staff) and heavy exposure to SARS-CoV-2 at work (categorized into two groups: “yes” or “no” according to answers to the question “Potential for heavy exposure to SARS-CoV-2 at work.”), number of household members (categorized into three groups: 0, 1–2, and ≥3), and close contact with patients with COVID-19, in addition to Model 1 variables.

The final multivariable model, Model 3, was further adjusted for chronic disease status, alcohol consumption, smoking status, infection prevention practices (wearing a mask and maintaining social distance), and vaccination status, in addition to Model 2 variables.

In consideration of previously reported findings (16), we performed an additional exploratory subgroup analysis stratified by the number of household members. This subgroup analysis aimed to control the potential confounding effect of household transmission and to investigate whether the relationship between commuting distance and infection risk differs among individuals with ≤2 versus ≥3 household members.

For categorical variables, we employed the chi-squared test, while for continuous variables, we utilized the Mann–Whitney U test. Significance tests were two-sided, and the significance level for all analyses was set at p < 0.05. We used the “statsmodels” package for generalized linear models using Python, version 3.9.13 (Python Software Foundation, Wilmington, DE, USA).

## RESULTS

### Participant characteristics

A total of 1,716 staff members, including full-time and part-time medical professionals, researchers, and administrative staff, were recruited. Of those, 1,206 participated in the study. Thirty participants with unavailable commuting information and 220 participants with incomplete data on adjustment variables were excluded. Data on a total of 956 participants were analyzed in the study.

The baseline characteristics of the participants, categorized by antibody status, are summarized in Table I. The study population comprised 956 individuals, of whom 12.3% (n = 118) were antibody-positive. Significant differences were observed across several variables between the antibody-positive and antibody-negative groups. The analysis showed significant differences in SARS-CoV-2 antibody status across different job exposure risk groups (p = 0.01). The antibody-positive group were more likely to report close contact with COVID-19 patients (37.3% versus 10.6%, p < 0.001). Additionally, participants in the antibody-positive group were more likely to reside in households with ≥3 members (43.2% versus 23.7%, p < 0.001). Other examined factors, including age, gender distribution, BMI, vaccination status, adherence to infection prevention practices, and chronic disease prevalence, did not show statistically significant differences between the antibody-positive and antibody-negative groups.

The antibody-positive rates of the participants, categorized based on commuting distance by public transportation, were 11.1% (n = 44), 11.9% (n = 35), and 14.6% (n = 39), respectively. The baseline characteristics of the participants are summarized in Table II. Among the participants, 560 (58.6%) used public transportation for commute (median 11.1, IQR [4.6, 20.7] km). The median (min–max) commuting distances using public transportation for the short and long commuting-distance groups were 4.7 (1.0–11.2) and 21.3 (11.3–97.9) km, respectively. The non-users of public transportation group (n = 396; mean age 38.1 years; 60.4% female) had the highest proportion of high-risk job types for COVID-19 exposure (61.9%) and heavy exposure to SARS-CoV-2 at work (30.6%). The short commuting-distance group (n = 293; mean age 35.0 years; 81.9% female) had the highest proportion of female participants. The long commuting-distance group (n = 267; mean age 40.0 years; 66.3% female) had the lowest proportion of high-risk occupations (39.7%) and heavy exposure to SARS-CoV-2 at work (16.9%). Across all groups, 515 (53.9%) participants with high-risk jobs for COVID-19 exposure were doctors or nurses, 924 (96.7%) participants wore a mask, 949 (99.3%) maintained social distance as infection prevention practices, and 934 (97.7%) participants received at least one dose of the COVID-19 vaccine.

### Commuting distance using public transportation and antibody-positivity risk

Table III summarizes the relationship between commuting distance using public transportation and antibody-positivity across the three different models. When adjusted for age, sex, and BMI, commuting distance by public transportation was not associated with antibody-positivity compared with commuting with no public transportation (short: adjusted OR 1.09 [95% CI 0.67–1.77; long: adjusted OR 1.42 [95% CI 0.89–2.27]). The results were similar when additionally adjusted for job type, heavy exposure to SARS-CoV-2 at work, number of household members, and close contact with patients with COVID-19 (short: adjusted OR 1.23 [95% CI 0.74–2.06]; long: adjusted OR 1.69 [95% CI 1.01–2.81]). The results were also similar when additionally adjusted for chronic diseases, alcohol consumption, smoking status, infection prevention practices, and vaccination status (short: adjusted OR 1.18 [95% CI 0.70–1.98]; long: adjusted OR 1.62 [95% CI 0.97–2.71]).

### Association between antibody-positivity and number of household members

The baseline characteristics of the participants, categorized by the number of household members, are summarized in Table IV. The participants were divided into two groups: those with ≤2 household members and those with ≥3 household members. Participants with ≤2 household members were younger and more likely to be female than those with ≥3 household members. Participants in the ≥3 household-members group were more likely to experience close contact with patients with COVID-19 (21.6% versus 11.2%) and have a higher antibody-positivity rate (20.4% versus 9.5%) than that of those in the ≤2 household-members group. In participants with ≤2 household members, compared to those with no use of public transportation, the odds of having a positive antibody was higher for those with a long commuting distance across all three models (Table V; Model 1: adjusted OR 2.01 [95% CI 1.04–3.89], Model 2: 2.01 [95% CI 1.04–3.89], and Model 3: 1.98 [95% CI 1.02–3.84]). Neither short nor long commuting distances using public transportation were associated with antibody-positivity in participants with ≥3 household members in all three models.

## DISCUSSION

Although it has been reported that over 40% of public transportation users switched to alternative commuting methods, such as private vehicles, walking, or remote work, after the COVID-19 outbreak (18), public transport was increasingly recognized as a potential factor for the spread of SARS-CoV-2, with several studies reporting elevated risks of virus transmission among users of buses and trains (8–11). Many of these studies depended on self-reported, internet-based surveys that may overlook asymptomatic infections, thus potentially underestimating the true spread of the virus. To address these limitations, our study utilized antibody testing against the N protein of the virus. This method detects previous infections, regardless of vaccination status and symptom presentation, over a period of several months. Additionally, our current research built upon existing findings related to SARS-CoV-2 infection, such as sex (19, 20), age (21), and occupation (22, 23), with the unique element of commuting distance using public transportation.

While Japanese internet-based questionnaire survey reported longer commuting distances were associated with lower infection rates (8), our study suggests that commuting distance does not contribute to increased infection risk in Japan. This discrepancy may be explained by the fact that we used the antibody-positive rate as an indicator to assess SARS-CoV-2 infection to include subclinical infection, which may reflect more accurate results from the questionnaire in the previous study. We noted that the same report highlighted that longer commuting time contributed to increased risk of SARS-CoV-2 infection (8), and that a study of infection risk model in China reported that ventilation and distance between commuters in subways could be also associated with increased risk (24). Since diverse factors can contribute to SARS-CoV-2 infection during commuting, building a commuting risk model that includes our results and previously reported factors would be recommended.

A unique aspect of this study involved conducting a subgroup analysis based on household size. This approach was taken based on previous research indicating that larger household size could significantly influence SARSCoV-2 infection dynamics (16). Individuals living in larger households may have a greater risk of being exposed to the virus through close contact with other household members, which may confound the association between commuting patterns and infection risk. Moreover, household size is often associated with commuting distance, as larger households may need to reside farther from workplaces to meet the needs of all members. By conducting a subgroup analysis based on household size, we aimed to account for the potential confounding effect of household transmission on the relationship between commuting distance and SARS-CoV-2 antibody-positivity. Interestingly, we found that the use of public transportation was associated with a likelihood of SARS-CoV-2 infection in individuals with ≤2 household members. On the contrary, such association was not found in the group of ≥3 household members.

Although household transmission of COVID-19 increases as the household size expands (25), our results suggested that the risk of infection during commuting is higher when the number of household members is small. We assumed that healthcare workers with larger households might likely exercise greater caution to prevent introducing infections into their families. A report in England suggests that household density affects infection risk (26), thus highlighting national characteristics like living environment would also help detailed investigation of infection risk during commuting, in addition to the investigation of the age groups of household members. The age structure of household members could potentially influence our findings, even though this variable was not included in the current study. For instance, teenagers are often considered a possible transmitter of many respiratory infectious diseases. Thus, further research would be needed on the differences in household age groups.

The limitation in this study is as follows: conducting a complete case analysis may have introduced selection bias by excluding participants with missing data. This exclusion may have led to a non-representative sample and may have overlooked the underlying mechanisms associated with the missing data. Additionally, we focused only on one hospital in Japan and thus, our findings might not be broadly generalizable. Furthermore, despite adjusting for several confounders, other variables not accounted for, such as the type of public transport used, the crowding of public transportation, or private activities, could also have influenced the results. Future research should aim to include a broader demographic and geographic sample and consider detailed tracking of individual commuting patterns and protective measures (27).

Identifying a high-risk group based on commuting distance using public transportation may help policymakers to design more targeted interventions. For example, increased sanitization efforts or more strict social distancing protocols could be implemented on public transport routes commonly used for longer commutes with ≤2 household members. In workplaces such as hospitals, understanding the risk posed by the number of household members in addition to commuting patterns could also allow employers to make informed decisions about flexible working arrangements or shuttle services for their staff.

In conclusion, our study found no overall association between commuting distance using public transportation and SARS-CoV-2 antibody-positivity. However, among healthcare workers with ≤2 household members, there was a significant association, suggesting that public transportation could play a crucial role in virus transmission within this specific group. These findings highlight the importance of targeted public health measures, such as enhanced sanitation and social distancing on public transport, to mitigate transmission risks.

## Figures and Tables

**Table I:** Characteristics and risk factors of SARS-CoV-2 antibody-positive versus negative participants

	Overall	Antibody-positive	Antibody-negative	P-values
No. of participants, n (%)	956 (100)	118 (12.3)	838 (87.7)	
Age, mean (SD), yr	37.7 (11.2)	36.4 (10.2)	37.9 (11.3)	0.26
Female sex, n (%)	656 (68.6)	77 (65.3)	579 (69.1)	0.46
BMI				0.77
Overweight, n (%)	141 (14.7)	20 (16.9)	121 (14.4)	
Healthy weight, n (%)	687 (71.9)	83 (70.3)	604 (72.1)	
Underweight, n (%)	128 (13.4)	15 (12.7)	113 (13.5)	
Exposure risk by job types				0.01
High-risk, n (%)	515 (53.9)	79 (66.9)	436 (52.0)	
Middle-risk, n (%)	188 (19.7)	19 (16.1)	169 (20.2)	
Low-risk, n (%)	253 (26.5)	20 (16.9)	233 (27.8)	
Heavy exposure to SARS-CoV-2 at work, n (%)	240 (25.1)	33 (28.0)	207 (24.7)	0.51
Close contact with patients with COVID-19	133 (13.9)	44 (37.3)	89 (10.6)	<0.001
≥3 household-members, n (%)	250 (26.2)	51 (43.2)	199 (23.7)	<0.001
One dose of COVID-19 vaccine, n (%)	934 (97.7)	117 (99.2)	817 (97.5)	0.43
Adherence to infection-prevention practices				
Maintaining social distance, n (%)	924 (96.7)	115 (97.5)	809 (96.5)	0.81
Wearing a mask, n (%)	949 (99.3)	118 (100.0)	831 (99.2)	0.67
Chronic disease, n (%)	168 (17.6)	18 (15.3)	150 (17.9)	0.56
Smoking, n (%)	37 (3.9)	5 (4.2)	32 (3.8)	0.99
Alcohol consumption, n (%)	606 (63.4)	83 (70.3)	523 (62.4)	0.12

SD, standard deviation; BMI, body mass index; SARS-CoV-2, severe acute respiratory syndrome coronavirus 2; COVID-19, coronavirus disease 2019.

Characteristics are presented with mean (SD) for continuous variables and numbers (%) for categorical variables.

**Table II:** Characteristics of eligible participants grouped by commuting distance using public transportation

		Commuting-distance groups using public transportation		
	
	Overall	Non-users of public transportation	Short commuters	Long commuters
Commuting distance using public transportation, median (min–max), km		0	4.7 (1.0–11.2)	21.3 (11.3–97.9)
No. of participants, n (%)	956 (100)	n = 396	n = 293	n = 267
Age, mean (SD), yr	37.7 (11.2)	38.1 (10.8)	35.0 (11.0)	40.0 (11.4)
Female sex, n (%)	656 (68.6)	239 (60.4)	240 (81.9)	177 (66.3)
BMI				
Overweight, n (%)	141 (14.7)	61 (15.4)	33 (11.3)	47 (17.6)
Healthy weight, n (%)	687 (71.9)	290 (73.2)	205 (70.0)	192 (71.9)
Underweight, n (%)	128 (13.4)	45 (11.4)	55 (18.8)	28 (10.5)
Exposure risk by job types				
High-risk, n (%)	515 (53.9)	245 (61.9)	164 (56.0)	106 (39.7)
Middle-risk, n (%)	188 (19.7)	68 (17.2)	53 (18.1)	67 (25.1)
Low-risk, n (%)	253 (26.5)	83 (21.0)	76 (25.9)	94 (35.2)
Heavy exposure to SARS-CoV-2 at work, n (%)	240 (25.1)	121 (30.6)	74 (25.3)	45 (16.9)
No. of household members				
≤2, n (%)	706 (73.8)	295 (75.5)	231 (78.8)	180 (67.4)
≥3, n (%)	250 (26.2)	101 (25.5)	62 (21.2)	87 (32.6)
Close contact with patients with COVID-19	133 (13.9)	60 (15.2)	40 (13.7)	33 (12.4)
One dose of COVID-19 vaccine, n (%)	934 (97.7)	390 (98.5)	284 (96.9)	260 (97.4)
Adherence to infection-prevention practices				
Maintaining social distance, n (%)	924 (96.7)	390 (98.5)	284 (96.9)	259 (97.0)
Wearing a mask, n (%)	949 (99.3)	381 (96.2)	293 (100.0)	266 (99.6)
Chronic disease, n (%)	168 (17.6)	67 (16.9)	48 (16.4)	53 (19.9)
Smoking, n (%)	37 (3.9)	18 (4.5)	12 (4.1)	7 (2.6)
Alcohol consumption, n (%)	606 (63.4)	240 (60.6)	192 (65.5)	174 (65.2)

SD, standard deviation; BMI, body mass index; SARS-CoV-2, severe acute respiratory syndrome coronavirus 2; COVID-19, coronavirus disease 2019.

Characteristics are presented with mean (SD) for continuous variables and numbers (%) for categorical variables.

**Table III:** Odds ratios (95% CI) and P-values for trend of antibody-positivity according to tertiles of commuting distance using public transportation

Commuting distance using public transportation, median (min–max), km	Commuting-distance groups using public transportation			P-values for trend

	Non-users of public transportation	Short commuters	Long commuters	
	0	4.7 (1.0–11.2)	21.3 (11.3–97.9)	
No. of participants	n = 396	n = 293	n = 267	
Model 1^a^	[Ref]	1.09 (0.67–1.77)	1.42 (0.89–2.27)	0.15
Model 2^b^	[Ref]	1.23 (0.74–2.06)	1.69 (1.01–2.81)	0.05
Model 3^c^	[Ref]	1.18 (0.70–1.98)	1.62 (0.97–2.71)	0.07

CI, confidence interval; BMI, body mass index.

a Model 1 is adjusted for age, sex, and BMI.

b Model 2 is adjusted for covariates of Model 1 + job types + heavy exposure + number of household members + close contact.

c Model 3 is adjusted for covariates of Model 2 + vaccine + adherence to preventive measures + presence of chronic disease + smoking status + alcohol consumption.

**Table IV:** Characteristics of eligible participants by number of household members

	Number of household members		P-values

	≤2	≥3	
No. of participants, n (%)	706 (73.8)	250 (26.2)	
Age, mean (SD), yr	36.8 (11.6)	40.1 (9.6)	<0.001
Female sex, n (%)	511 (72.4)	145 (58.0)	<0.001
BMI			0.64
Overweight, n (%)	100 (14.2)	41 (16.4)	
Healthy weight, n (%)	509 (72.1)	178 (71.2)	
Underweight, n (%)	97 (13.7)	31 (12.4)	
Exposure risk by job types			0.64
High-risk, n (%)	382 (54.1)	133 (53.2)	
Middle-risk, n (%)	134 (19.0)	54 (21.6)	
Low-risk, n (%)	190 (26.9)	63 (25.2)	
Heavy exposure to SARS-CoV-2 at work, n (%)	185 (26.2)	45 (16.9)	0.01
Close contact with patients with COVID-19	79 (11.2)	54 (21.6)	<0.001
One dose of COVID-19 vaccine, n (%)	689 (97.6)	245 (98.0)	0.90
Adherence to infection-prevention practices			
Maintaining social distance, n (%)	680 (96.3)	244 (97.6)	0.44
Wearing a mask, n (%)	700 (99.2)	249 (99.6)	0.76
Chronic disease, n (%)	123 (17.4)	53 (19.9)	0.22
Smoking, n (%)	23 (3.3)	14 (5.6)	0.14
Alcohol consumption, n (%)	448 (63.5)	158 (63.2)	0.99
Antibody-positive, n (%)	67 (9.5)	51 (20.4)	<0.001

SD, standard deviation; BMI, body mass index; SARS-CoV-2, severe acute respiratory syndrome coronavirus 2; COVID-19, coronavirus disease 2019.

Characteristics are presented with mean (SD) for continuous variables and numbers (%) for categorical variables.

**Table V:** Odds ratios (95% CI) and P-values for trend of antibody-positivity according to tertiles of public transportation commuting distance: Subgroup analysis by number of household members

Commuting distance using public transportation, median (min–max), km	Commuting-distance groups using public transportation			P-values for trend

	Non-users of public transportation	Short commuters	Long commuters	
	0	4.7 (1.0–11.2)	21.3 (11.3–97.9)	
	**Living in households with ≤2 members**			
No. of participants	n = 295	n = 231	n = 180	
Model 1^a^	[Ref]	1.22 (0.65–2.28)	1.89 (1.00–3.57)	0.05
Model 2^b^	[Ref]	1.24 (0.65–2.35)	2.01 (1.04–3.89)	0.04
Model 3^c^	[Ref]	1.19 (0.62–2.27)	1.98 (1.02–3.84)	0.05

	**Living in households with ≥3 members**			
No. of participants	n = 101	n = 62	n = 87	
Model 1^a^	[Ref]	0.92 (0.45–1.90)	0.90 (0.39–2.05)	0.82
Model 2^b^	[Ref]	1.54 (0.59–4.03)	1.48 (0.64–3.44)	0.35
Model 3^c^	[Ref]	1.29 (0.53–3.10)	1.50 (0.57–3.99)	0.57

CI, confidence interval; BMI, body mass index.

a Model 1 is adjusted for age, sex, and BMI.

b Model 2 is adjusted for covariates of Model 1 + job types + heavy exposure + close contact.

c Model 3 is adjusted for covariates of Model 2 + vaccine + adherence to preventive measures + presence of chronic disease + smoking status + alcohol consumption.
